# Explainable statistical learning in public health for policy development: the case of real-world suicide data

**DOI:** 10.1186/s12874-019-0796-7

**Published:** 2019-07-17

**Authors:** Paul van Schaik, Yonghong Peng, Adedokun Ojelabi, Jonathan Ling

**Affiliations:** 1 School of Social Sciences, Humanities and Law, Teesside University, Borough Road, Middlesbrough, TS1 3BA UK; 2 The University of Sunderland, St Peters Campus, St Peters Way, Sunderland, SR6 0DD UK

**Keywords:** Public health data, Mental health, Suicide, Statistical learning, Machine learning, Explainability, Interpretability, Feature reduction, Health informatics

## Abstract

**Background:**

In recent years, the availability of publicly available data related to public health has significantly increased. These data have substantial potential to develop public health policy; however, this requires meaningful and insightful analysis. Our aim is to demonstrate how data analysis techniques can be used to address the issues of data reduction, prediction and explanation using online available public health data, in order to provide a sound basis for informing public health policy.

**Methods:**

Observational suicide prevention data were analysed from an existing online United Kingdom national public health database. Multi-collinearity analysis and principal-component analysis were used to reduce correlated data, followed by regression analyses for prediction and explanation of suicide.

**Results:**

Multi-collinearity analysis was effective in reducing the indicator set of predictors by 30% and principal component analysis further reduced the set by 86%. Regression for prediction identified four significant indicator predictors of suicide behaviour (emergency hospital admissions for intentional self-harm, children leaving care, statutory homelessness and self-reported well-being/low happiness) and two main component predictors (relatedness dysfunction, and behavioural problems and mental illness). Regression for explanation identified significant moderation of a well-being predictor (low happiness) of suicide behaviour by a social factor (living alone), thereby supporting existing theory and providing insight beyond the results of regression for prediction. Two independent predictors capturing relatedness needs in social care service delivery were also identified.

**Conclusions:**

We demonstrate the effectiveness of regression techniques in the analysis of online public health data. Regression analysis for prediction and explanation can both be appropriate for public health data analysis for a better understanding of public health outcomes. It is therefore essential to clarify the aim of the analysis (prediction accuracy or theory development) as a basis for choosing the most appropriate model. We apply these techniques to the analysis of suicide data; however, we argue that the analysis presented in this study should be applied to datasets across public health in order to improve the quality of health policy recommendations.

**Electronic supplementary material:**

The online version of this article (10.1186/s12874-019-0796-7) contains supplementary material, which is available to authorized users.

## Background

Using complex datasets to generate insight into a range of health and public health concerns is of particular interest and importance to public health organisations in order to develop strategies to improve the health of the public [10, 14], for example through determining which indicators are genuinely important predictors of outcomes of interest. The results of statistical learning^1^ analysis of complex data can be overwhelming for end users, such as those developing public health strategies at a local level. Therefore, data reduction methods may be desirable to reduce information overload for users. The analysis of complex data can also help indicate which data are missing or could be used to replace other data sources, and potentially save the cost of time-consuming or expensive data collection. Furthermore, complex data can also be used to generate new insights, for instance through facilitating comparisons to establish how indicators or local authorities cluster for specific conditions.

One difficulty with complex data is that users may lack the skills to extract meaning from them [16, 37]. Skills gaps in the interpretation of complex data by public health analysts could therefore lead to the development of suboptimal strategies, potentially diverting scarce resources away from areas where they may have a greater impact on health [2, 23]. Public health analysts need to build confidence in the use of inferential data analysis methods and interpreting the results. For example, if different models are run on the same data they may generate different sets of important predictor variables [37], which has potential consequences for strategy development and ultimately sub-optimal allocation of resources. This is an inevitable consequence of predictive research, in which inferences can only be made about the combination of variables that best predict an outcome, given specific variable selection procedures and constraints imposed by the researcher [20, 32]. While analysts are likely to be aware of the wide range of analysis techniques available, they may be less sure when to use which techniques or how to combine these. Similarly, in dimension reduction analyses they may be uncertain about how to choose the number of higher-order variables (clusters/factors).

Two different perspectives have implications for variable selection in the statistical modelling of outcomes; these are, first, prediction and, second, explanation ([32], pp. 195–197; [33], pp. 305–308; [37, 41]) or inference ([20], pp. 17–20). These perspectives are roughly equivalent to Breiman’s [5] distinction between ‘the data-modelling culture’ and the ‘algorithmic-modelling culture’. The aim of predictive research is to develop models to predict outcome variables that are of interest from a set of predictors. By contrast, the aim of explanatory research is to test hypotheses regarding phenomena of interest. These two perspectives are not necessarily mutually exclusive. For example, in predictive research variable selection may be based on theory. Otherwise, the designation of particular variables as predictors or outcomes may be based on practical considerations. Moreover, in less-developed areas or in applied work, when prediction is feasible, explanations are not always possible or can be vague [33]. Crucially, serious threats to validity from model misspecification in explanatory research do not apply to predictive research [33]. Despite potential overlap, it is usually straightforward to identify studies and research where the main purpose is either prediction or explanation [20, 32].

In public health literature both research perspectives are represented, as the following examples show. Predictive research has used stepwise multiple regression procedures to predict life expectancy in a highly polluted environment [38], and used multiple regression models to predict waist circumference from body mass index [4]. Analysis for prediction through stepwise regression and other methods has also been used to predict methicillin-resistant *Staphylococcus aureus* (MRSA) carriage in surgical wards [25].

Explanatory research has developed a model to explain variation in longevity from health expenditure in Organisation for Economic Co-operation and Development (OECD) countries and tested the model using multiple regression [1]. Furthermore, a model of successful implementation of pharmaceutical services was developed, based on the theory of planned behaviour, and tested with multiple regression analysis and mediation analysis [44]. Logistic regression has also been used to investigate whether breastfeeding at infancy was associated with reduction in risk of obesity and mental health at 9 years of age [36].

Considering these two research perspectives, we undertook the current study using national data available for suicides in England. We focused on suicide, as the causes of suicide are complex [39]; with a better understanding of the data on suicide, better strategies for prevention can be developed and implemented [31, 47, 48].

Two main issues in data analysis on this topic include (i) reducing sets of correlated indicators [28], and (ii) predicting important public health outcomes [6]. The aim of this paper is to demonstrate how data analysis techniques can be used to address the issues of data reduction, prediction and explanation using online public health data, in order to provide a sound basis for informing public health policy. This work contributes to the field by providing guidance on the analysis of public health data.

Accordingly, we address the following questions:

- *Question 1:* What advice can be given to public health analysts to reduce sets of correlated public health data?
- *Question 2:* What advice can be given to aid decisions related to selecting predictors for importance?
- *Question 3:* What advice can be given to decide regarding the trade-off between predictive power and interpretability?

## Methods

Given the aim of demonstrating the use of data analysis techniques for data reduction of indicator variables (features), and prediction and explanation of outcome variables (labels), we analysed observational national public health data.

### Data preparation

In this work we used Fingertips (https://fingertips.phe.org.uk/) which is a large repository of indicator aggregate UK public health data. The unit of analysis is organisation (e.g., local authority). Data are structured by thematic profiles, with more than 1600 indicators across more than 60 profiles.^2^ Data are visualised through a visualisation platform as part of the repository, but are also publicly available via an API (https://fingertips.phe.org.uk/api). The main target users are UK local authorities, National Health Service (NHS) (service commissioners mainly) and policymakers (such as Public Health England [PHE]).

We extracted the suicide profile data using fingertipsR [12]. The extracted data set represents the population of England (> 54,000,000). We then selected local authority (county and unitary authority) as the level of data aggregation.^3^ We used three different methods to impute missing values for different indicators: (1) calculating (count × denominator)/100000 where available, (2) otherwise the indicator mean of the remaining authorities without missing values in the same region, (3) if data were sparse (count < 10), the median value.

Franklin et al. [13] explain that, in any meaningful data analysis that aims to establish suicide predictors, the measurement of predictors must precede the measurement of the outcome (suicide) data. Franklin et al. also established that there is a lack of studies analysing the combined effect of multiple risk factors on suicide (although this has recently become an increasing area of focus, particularly in the USA; see [42]). Accordingly, we selected the most recent outcome data (2014 suicide rate) for analysis. We then analysed all available suicide predictor variables from the most recent previous years (2013 where available, otherwise 2012 or, failing that, 2011). Table 1 presents descriptives of the outcome measure (suicide rate) and its potential predictors.

**Table 1:** Descriptives of outcome measure and predictor variables (*N* = 149)

	Minimum	Maximum	Mean	*SD*
2014 Suicide (age-standardised rate per 100,000 - outcome measure)	6.12	18.26	10.13	2.14
2013 Adult social care users who have as much social contact as they would like (% of adult social care users)	35.40	54.40	43.89	3.98
2013 Adults in treatment at specialist alcohol misuse services (rate per 1000 population)	0.67	6.19	2.40	1.08
2013 Adults in treatment at specialist drug misuse services (rate per 1000 population)	1.69	16.07	5.59	2.48
2013 Alcohol-related hospital admission (female) (directly standardised rate per 100,000 female population)	498.46	1386.28	903.85	175.78
2013 Alcohol-related hospital admission (male) (directly standardised rate per 100,000 male population)	1011.15	2819.52	1805.52	344.23
2013 Alcohol-related hospital admission (directly standardised rate per 100,000 population)	731.04	2069.94	1318.11	253.33
2013 Children in the youth justice system (rate per 1,000 aged 10–18)	2.91	17.08	7.75	2.77
2013 Children leaving care (rate per 10,000 < 18 population)	9.59	70.62	28.78	10.32
2013 Depression recorded prevalence (% of adults with a new diagnosis of depression who had a bio-psychosocial assessment)	3.09	10.71	6.48	1.40
2013 Domestic abuse incidents (rate per 1,000 population)	4.87	30.38	19.84	4.74
2013 Emergency hospital admissions for intentional self-harm (female) (directly age-standardised rate per 100,000 women)	76.46	751.06	257.43	103.75
2013 Emergency hospital admissions for intentional self-harm (male) (directly age-standardised rate per 100,000 men)	45.43	614.41	166.45	81.79
2013 Emergency hospital admissions for intentional self-harm (directly age-and-sex-standardised rate per 100,000)	60.23	682.62	211.02	90.47
2013 Looked after children (rate per 10,000 < 18 population)	19.83	153.29	64.91	25.10
2013 Self-reported well-being - high anxiety (% of people)	9.61	29.71	20.27	2.75
2013 Severe mental illness recorded prevalence (% of practice register [all ages])	0.47	1.47	0.87	0.19
2013 Social care mental health clients receiving services (rate per 100,000 population)	67.43	2331.12	387.68	299.36
2013 Statutory homelessness (rate per 1000 households)	0.10	12.55	2.54	2.18
2013 Successful completion of alcohol treatment (% who do not represent within 6 months)	15.13	67.59	37.40	8.79
2013 Successful completion of drug treatment - non-opiate users (% who do not represent within 6 months)	7.08	59.72	36.95	8.60
2013 Successful completion of drug treatment - opiate users (% who do not represent within 6 months)	3.52	15.79	8.15	2.41
2013 Unemployment (% of working-age population)	3.70	14.50	7.99	2.52
2012 Adult carers who have as much social contact as they would like (18+ yrs) (% of 18+ carers)	23.90	58.50	40.95	7.24
2012 Adult carers who have as much social contact as they would like (all ages) (% of adult carers)	23.90	58.50	40.95	7.24
2011 Estimated prevalence of opiates and/or crack cocaine use (rate per 1,000 aged 15–64)	2.93	20.76	9.13	3.79
2011 Long-term health problems or disability (% of people whose day-to-day activities are limited by their health or disability)	11.20	25.57	17.68	3.26
2011 Marital breakup (% of adults whose current marital status is separated or divorced)	7.73	16.30	11.67	1.24
2011 Older people living alone (% of households occupied by a single person aged 65 or over)	2.29	7.57	5.12	1.06
2011 People living alone (% of all households occupied by a single person) Mental Health Service users with crisis plans: % of people in contact with services with a crisis plan in place (end of quarter snapshot) Older people	8.02	23.42	13.03	2.20
2011 Self-reported well-being - low happiness (% of people with a low happiness score)	6.55	17.68	10.98	2.09

### Data analysis

We conducted the following analyses: graphical and numerical examination of variable distribution and transformation (see Additional files 1 and 3), multicollinearity analysis, principal component analysis (both to answer Question 1; see Section 3.1), and stepwise, hierarchical and standard regression (all three to answer Question 2; see Section 3.2 and Additional file 2).

### Availability of data and materials

The dataset and the data analysis are available at http://sssl-staffweb.tees.ac.uk/u0011128/Fingertips.data.and.analysis/R.zip and http://sssl-staffweb.tees.ac.uk/u0011128/Fingertips.data.and.analysis/SPSS.zip. The dataset can also be accessed via the R package fingertipsR (https://cran.r-project.org/web/packages/fingertipsR/index.html).

## Results

We address each of the three questions with an analysis of the Fingertips suicide data in the following three sections.

### Reducing the set of indicators

Before the variables in a data set are reduced in preparation for regression analysis, all interval and ratio variables are first screened for normality and transformed to achieve this where normality does not exist [43] (see Additional file 1).

Two approaches can be distinguished to reduce sets of correlated data: (1) multicollinearity analysis to remove (a subset of) existing variables and (2) principal-component analysis to replace the existing variables by new higher-order variables.

#### Multi-collinearity analysis

We illustrate Approach 1 by analysing the predictor variables of suicide. The aim is to reduce the set of predictor variables by identifying, diagnosing and removing problematic potential predictors that suffer from multi-collinearity. By definition, problematic variables can be predicted to a large extent or fully from one or more other predictors [20, 43].

*Identification* of problematic predictors is achieved by conducting regression analysis with each predictor variable as the outcome variable and the remaining variables as predictors, and noting the tolerance statistic or the variance inflation factor (VIF) statistic. Cut-off values are then applied beyond which variables are identified as problematic: < 0.1 or < 0.2 (for tolerance) and > 10 or > 5 (for VIF) [43]. Table 2 presents the multi-collinearity statistics tolerance and VIF for each predictor. Using the cut-offs for multi-collinearity, we noted 13 out of 30 predictors that suffered from multi-collinearity.

**Table 2:** Multi-collinearity analysis

Variable #	Variable name	Transformation^a^	Tolerance	VIF	Decision
2	Adult social-care users who have as much social contact as they would like	–	0.64	1.57	Keep
3	Adults in treatment at specialist alcohol misuse services	Log	0.31	3.24	Keep
4	Adults in treatment at specialist drug misuse services	Log	0.08	12.66	Remove
5	Alcohol-related hospital admission (female)	Log	0.00	422.64	Remove
6	Alcohol-related hospital admission (male)	Log	0.00	1126.14	Remove
7	Alcohol-related hospital admission (all)	Log	0.00	2813.20	Keep
8	Children in the youth justice system	Log	0.36	2.74	Keep
9	Children leaving care	Log	0.22	4.56	Keep
10	Depression recorded prevalence	–	0.39	2.59	Keep
11	Domestic abuse incidents	–	0.49	2.03	Keep
12	Emergency hospital admissions for intentional self-harm (female)	square root	0.08	13.18	Remove
13	Emergency hospital admissions for intentional self-harm (male)	square root	0.06	17.53	Remove
14	Emergency hospital admissions for intentional self-harm (all)	square root	0.00	11956.59	Keep
15	Looked after children	Log	0.19	5.35	Remove
16	Self-reported well-being - high anxiety	–	0.65	1.53	Keep
17	Severe mental illness recorded prevalence	Log	0.30	3.30	Keep
18	Social care mental health clients receiving services	Log	0.75	1.34	Keep
19	Statutory homelessness	Log	0.41	2.41	Keep
20	Successful completion of alcohol treatment	–	0.45	2.22	Keep
21	Successful completion of drug treatment - non-opiate users	–	0.41	2.47	Keep
22	Successful completion of drug treatment - opiate users	Log	0.49	2.02	Keep
23	Unemployment	Log	0.16	6.40	Keep
24	Adult carers who have as much social contact as they would like (18+ yrs)	–	0.00	infinity	Remove
25	Adult carers who have as much social contact as they would like (all ages)	–	0.59	1.71	Keep
26	Estimated prevalence of opiates and/or crack cocaine use	Log	0.09	10.60	Keep
27	Long-term health problems or disability	–	0.08	12.84	Remove
28	Marital breakup	–	0.39	2.59	Keep
29	Older people living alone	–	0.08	11.77	Remove
30	People living alone	Inverse	0.23	4.36	Keep
31	Self-reported well-being - low happiness	–	0.36	2.77	Keep

^a^See Additional file 1

*Diagnosis* is carried out by analysing problematic predictors. For this purpose, the following were used: variance proportions of eigenvalues from the multiple regression used for identification, and stepwise regressions (including correlations) each time with a problematic predictor as outcome variable and the remaining potential predictors as predictors.

Of the predictors, *adults in treatment at specialist drug misuse services* and *estimated prevalence of opiates and/or crack cocaine use* were highly correlated (*r* = 0.91) and both shared their highest variance proportion with Eigenvalue 25. *Alcohol-related hospital admission* variables (female/male/all) were highly correlated (*r* ≥ 0.97) and shared their highest variance proportion with Eigenvalue 31. *Emergency hospital admission* variables *for intentional self-harm* (female/male/all) were substantially correlated (*r* ≥ 0.92) and shared their highest variance proportion with Eigenvalue 26. *Looked after children* was most highly correlated with *children leaving care* (*r* = 0.77) and both shared among their highest variance proportions with Eigenvalue 23. *Unemployment* was highly correlated with several other variables (most notably *adults in treatment at specialist drug misuse services*, *alcohol-related hospital admission* [female/male/all], and *estimated prevalence of opiates and/or crack cocaine use*; 0.70 ≤ *r* ≤ 0.73) and shared its highest proportion of variance with Eigenvalue 27, as did *long-term health problems or disability*. *Adult carers who have as much social contact as they would like* (aged 18 or above) suffered from singularity (tolerance = 0/VIF = infinity). *Estimated prevalence of opiates and/or crack cocaine use* was highly correlated with *unemployment* (*r* = 0.71) and shared its highest proportion of variance with Eigenvalue 25, as did *adults in treatment at specialist drug misuse services*. *Long-term health problems or disability* was highly correlated with *emergency hospital admissions for intentional self-harm* (male) (*r* = 0.73) and *marital breakup* (*r* = 0.70) and substantially correlated with seven other variables (*r* > 0.50) and shared its highest proportion of variance with Eigenvalue 26, as did *emergency hospital admissions for intentional self-harm* (female/male). *Older people living alone* was highly correlated with *long-term health problems or disability*, but also substantially correlated with *emergency hospital admissions for intentional self-harm female/male/all* (0.51 ≤ *r* ≤ 0.55) and *people living alone* (*r* = − 0.45).

*Removal* is performed by evaluating and acting on the diagnostic information, considering both statistical and theoretical considerations. We removed nine problematic variables. In particular, we replaced *adults in treatment at specialist drug misuse services* with *estimated prevalence of opiates and/or crack cocaine use* because, first, they were highly correlated and, second, latter is a behavioural measure, while former is a measure of treatment received and therefore arguably a consequent of the latter rather than a cause. We kept *alcohol-related hospital admissions (all)* and removed the female and male counterparts because the overall admission rate represents the whole population (both male and female) and is therefore more generally applicable. Similarly, we kept *emergency hospital admissions for intentional self-harm (all)* and removed the sex-specific counterparts. We replaced *looked-after children* with *children leaving care* because the two were highly correlated, but the latter did not suffer from multi-collinearity. We removed *adult carers who have as much social contact as they would like (age 18 or above)* because it suffered from singularity. We removed *long-term health problems or disability*, as it was highly or substantially correlated with nine other variables. We removed *older people living alone* because of its substantial correlations with several other variables, including *people living alone*, which applies to the whole population rather than specifically to older people.

With the nine variables that were selected for removal removed, we ran multi-collinearity analysis again. No further variables having multi-collinearity were identified.

#### Principal-component analysis

Principal-component analysis (PCA) is an unsupervised-learning technique to summarise a larger number of variables into a smaller number of higher-order components [20]. PCA can be a useful technique for public health policy makers because it allows the reduction of a potentially vast set of (predictor) indicators to a conceptually manageable and meaningful set of higher-order public health dimensions (such as relationship dysfunction). Note that PCA is only useful to the extent that the results can be understood by public health experts (rather than data scientists) and, because the results cannot be predicted in advance, the usefulness of the results cannot be guaranteed.

In the main analyses of PCA (or factor analysis), the first step is to determine the number of factors to extract. As a second step, it is then normally necessary to rotate an initial factor solution to produce interpretable results. In the third step, an interpretation of the rotated solution is made, if possible.

We ran PCA (Table 3) first, on the full set of potential predictors and, second, on the reduced set, with problematic variables removed after multi-collinearity analysis. We find the results of the second set of analyses more informative because the components are not influenced by highly correlated subsets of variables that were eliminated based on the results of multi-collinearity analysis. In the second set of analyses, based on Kaiser’s criterion (components/factors with eigenvalues ≥1 to be retained) five components should be extracted. However, based on parallel analysis three components should be extracted. The three-component PCA solution was by definition more concise and was more amenable to interpretation. The three-component solution explained 54% of variance in the variables entered into the PCA. We interpret Component 1 as behavioural problems and mental illness, Component 2 as relatedness dysfunction and Component 3 as success of addiction treatment. We saved the component scores from the three-component solution for use in subsequent analysis to predict suicide.

**Table 3:** Principal-component analysis: pattern matrix

	Component		
	1	2	3
Unemployment	**0.87**	−0.19	0.01
Estimated prevalence of opiates and/or crack cocaine use	**0.86**	−0.04	− 0.08
Alcohol-related hospital admission	**0.83**	0.12	0.06
Children leaving care	**0.82**	− 0.09	− 0.03
Severe mental illness recorded prevalence	**0.75**	−0.37	− 0.03
Self-reported well-being - low happiness	**0.71**	0.11	0.21
Children in the youth justice system	**0.69**	0.07	−0.15
Adults in treatment at specialist alcohol misuse services	**0.63**	0.32	−0.06
inverse People living alone	**−0.48**	−0.21	0.19
Self-reported well-being - high anxiety	**0.48**	−0.01	0.21
Domestic abuse incidents	**0.46**	0.07	−0.02
Emergency hospital admissions for intentional self-harm	0.25	**0.76**	0.03
Depression recorded prevalence	0.04	**0.76**	−0.03
Statutory homelessness	0.22	**−0.76**	−0.04
Marital breakup	0.20	**0.61**	0.10
Adult carers who have as much social contact as they would like (all ages)	−0.07	**0.56**	0.04
Adult social-care users who have as much social contact as they would like	0.00	**0.52**	−0.11
Successful completion of drug treatment - non-opiate users	0.12	0.08	**0.89**
Successful completion of alcohol treatment	−0.04	0.18	**0.80**
Successful completion of drug treatment - opiate users	−0.09	−0.27	**0.64**

In an initial three-factor solution, social care mental health clients receiving services loaded on none of the components. Therefore this variable was removed from the final analysis

Figures in bold indicate main loadings

### Analysing the importance of predictors

Two approaches to predictor selection in the supervised modelling of outcomes from predictors can be distinguished through regression analysis: prediction and explanation or inference [20, 32, 41].

#### Prediction research perspective: indicators as predictors

Under the prediction perspective, a prediction model is created, based on statistical criteria (e.g., variance explained). The selection of model predictors will be subject to sampling variation (e.g., from one annual sample to the next) and also variation caused by small differences between statistical algorithms. Therefore, the models are less likely to be generalisable across samples [11]; in other words, models are more likely to not to generalise between public health data sets. For example, after a few years of highly accurate predictions of flu incidence, Google Flu Trends overpredicted medical consultations for flu-like symptoms by 100% [15]. Cross-validation within or between samples then becomes important to ascertain the generalisability of models as an important criterion for model selection.

When data analysis is through multiple regression, a potential problem is that if all predictors under consideration are entered into a regression model then many of these may be statistically non-significant. This method is called ‘forced entry’ and is used in standard multiple regression. Common alternative methods to address this problem are forward, backward and stepwise selection. In forward entry, at each step the statistically significant predictor with the highest semi-partial correlation is selected until no more statistically significant predictors remain. In backward entry, at each step the statistically non-significant predictor with the lowest semi-partial correlation is removed until no more statistically non-significant predictors remain. Stepwise entry is a combination of the two previous methods. As in the forward method, at each step the statistically significant predictor with the highest semi-partial correlation is selected, but, as in the backward method, at each step the statistically non-significant predictor with the lowest semi-partial correlation is removed.

We illustrate the application of the prediction perspective to supervised modelling using stepwise multiple regression, with suicide rate as the outcome variable and the remaining predictors after multi-collinearity analysis. The statistically significant final model (Table 4) explained 44% (*R*^2^ = 0.44), *F* (4, 144) = 27.89, *p* < 0.001. The sample results overestimated the explained variance in the outcome by 2% (*R*^2^_adjusted_ = 0.42).^4^ The significant predictors were emergency hospital admissions for intentional self-harm, children leaving care, statutory homelessness and self-reported well-being/low happiness. These predictors were statistically significant at the point where they were entered into the regression, so each explained significant additional variance (*sr*^2^)^5^ in suicide rate over and above the previous predictors at their point of entry (Table 4). We address bias in regression modelling in Additional file 2. Additional file 3 presents a comparison of model results with and without transformations.

**Table 4:** Stepwise multiple regression

Source	Unstan-dardised coefficients		Stan-dardised coefficients	*F/t*	*p*	95%-confidence interval for *b*		Correlations			Collinearity statistics	
	*b*	*SE*	β			Lower limit	Upper limit	*r*	*sr*	*sr* ^*2*^	Tolerance	VIF
Model 1				64.07	< 0.001					0.30		
Constant	1.77	0.07		26.75	< 0.001	1.64	1.91					
Emergency hospital admissions for intentional self-harm: all^a^	0.04	0.00	0.55	8.00	< 0.001	0.03	0.05	0.55	0.55	0.55	1.00	1.00
Model 2				17.04	< 0.001					0.07		
Constant	1.30	0.13		9.92	< 0.001	1.04	1.56					
Emergency hospital admissions for intentional self-harm: all^a^	0.03	0.00	0.47	6.93	< 0.001	0.02	0.04	0.55	0.45	0.21	0.92	1.09
Children leaving care^b^	0.17	0.04	0.28	4.13	< 0.001	0.09	0.25	0.41	0.27	0.07	0.92	1.09
Model 3				9.09	< 0.001					0.04		
Constant	1.29	0.13		10.06	< 0.001	1.03	1.54					
Emergency hospital admissions for intentional self-harm: all^a^	0.02	0.01	0.36	4.63	< 0.001	0.01	0.03	0.55	0.29	0.09	0.69	1.46
Children leaving care^b^	0.21	0.04	0.36	5.07	< 0.001	0.13	0.30	0.41	0.32	0.10	0.79	1.27
Statutory homelessness^b^	−0.06	0.02	−0.23	−3.01	.003	−0.09	− 0.02	− 0.30	− 0.19	0.04	0.71	1.41
Model 4				5.98	< 0.001					0.02		
Constant	1.30	0.13		10.36	< 0.001	1.05	1.55					
Emergency hospital admissions for intentional self-harm: all^a^	0.03	0.01	0.40	5.13	< 0.001	0.02	0.04	0.55	0.32	0.10	0.65	1.53
Children leaving care^b^	0.26	0.04	0.43	5.69	< 0.001	0.17	0.34	0.41	0.36	0.13	0.68	1.47
Statutory homelessness^b^	−0.05	0.02	−0.21	−2.88	.005	−0.09	− 0.02	− 0.30	− 0.18	0.03	0.71	1.41
Self-reported well-being - low happiness	−0.02	0.01	−0.18	−2.44	.016	−0.03	0.00	0.13	−0.15	0.02	0.74	1.34

*R*^2^ = 0.44. *R*^2^_adjusted_ = 0.42

^a^square root-transformed

^b^log-transformed

#### Prediction approach: principal components as predictors

Using the three principal components from the previous PCA as predictors, we ran a further stepwise regression. The statistically significant final model (Table 5) explained 33% of variance in suicide rate (*R*^2^ = 0.33), *F* (2, 146) = 35.73, *p* < 0.001. The sample results overestimated the explained variance by 1% (*R*^2^_adjusted_ = 0.32). The significant positive predictors were Component 2 (relatedness dysfunction) and Component 1 (behavioural problems and mental illness). These predictors were statistically significant at the point where they were entered into the regression, so each explained significant additional variance (*sr*^2^) in suicide rate over and above the previous predictors at their point of entry (Table 6).

**Table 5:** Stepwise multiple regression on component score*s*

Source	Unstan-dardised coefficients		Stan-dardised coefficients	*F/t*	*p*	95%-confidence interval for *b*		Correlations			Collinearity statistics	
	*b*	*SE*	β			Lower limit	Upper limit	*r*	*sr*	*sr* ^*2*^	Tolerance	VIF
Model 1				44.70985	< 0.001					0.23		
Constant	2.29	0.01		155.42	< 0.001	2.26	2.32					
Component 2: relatedness dysfunction	0.10	0.01	0.48	6.69	< 0.001	0.07	0.13	0.48	0.48	0.48	1.00	1.00
Model 2				20.74	< 0.001					0.10		
Constant	2.29	0.01		165.53	< 0.001	2.27	2.32					
Component 2: relatedness dysfunction	0.09	0.01	0.43	6.32	< 0.001	0.06	0.12	0.48	0.43	0.18	0.98	1.03
Component 1: behavioural problems and mental illness	0.06	0.01	0.31	4.55	< 0.001	0.04	0.09	0.38	0.31	0.10	0.98	1.03

*R*^2^ = 0.33. *R*^2^_adjusted_ = 0.32

**Table 6:** Hierarchical multiple regression

Source	Unstan-dardised coefficients		Stan-dardised coefficients	*F/t*	*p*	95%-confidence interval for *b*		Correlations			Collinearity statistics	
	*b*	*SE*	β			Lower limit	Upper limit	*r*	*sr*	*sr* ^*2*^	Tolerance	VIF
Model 1				27.89	< 0.001							
Constant	1.30	0.13		10.36	< 0.001	1.05	1.55					
Emergency hospital admissions for intentional self-harm: all^a^	0.03	0.01	0.40	5.13	< 0.001	0.02	0.04	0.55	0.32	0.10	0.65	1.53
Children leaving care^b^	0.26	0.04	0.43	5.69	< 0.001	0.17	0.34	0.41	0.36	0.13	0.68	1.47
Statutory homelessness^b^	−0.05	0.02	−0.21	−2.88	.005	−0.09	−0.02	−0.30	−0.18	0.03	0.71	1.41
Self-reported well-being - low happiness	−0.02	0.01	−0.18	−2.44	.016	− 0.03	0.00	0.13	−0.15	0.02	0.74	1.34
				23.61	< 0.001					0.02		
Constant	1.33	0.13		10.63	< 0.001	1.09	1.58					
Emergency hospital admissions for intentional self-harm: all^a^	0.03	0.01	0.39	5.12	< 0.001	0.02	0.04	0.55	0.32	0.10	0.65	1.53
Children leaving care^b^	0.24	0.04	0.41	5.49	< 0.001	0.16	0.33	0.41	0.34	0.12	0.67	1.48
Statutory homelessness^b^	−0.02	0.01	−0.17	−2.44	.016	−0.03	0.00	0.13	−0.15	0.02	0.74	1.34
Self-reported well-being - low happiness	−0.04	0.02	−0.18	−2.32	.022	−0.08	−0.01	− 0.30	−0.14	0.02	0.66	1.51
Homelessness by low happiness	−0.01	0.01	−0.13	−2.03	.045	−0.03	0.00	−0.28	−0.13	0.02	0.90	1.11

*R*^2^ = 0.45. *R*^2^_adjusted_ = 0.43

^a^square root-transformed

^b^log-transformed

#### Explanatory approach: theory-based model

The explanatory approach uses theory to decide a priori on the predictors to include in a model and their order. Variables that theoretically are causal antecedents of the outcome variable are considered. When data analysis is through multiple regression, this approach uses hierarchical or forced entry of predictors. In forced entry all predictors are regressed onto the outcome variable simultaneously. In hierarchical entry, a set of nested models is tested, where each more complex model includes all the predictors of the simpler models; each model and its predictors are tested against a constant-only model (without predictors), and each model (except the simplest model) is tested against the most complex simpler model.

Here, we illustrate the explanatory approach, based on the hypothesis that environmental factors (e.g. living circumstances, such as homelessness) moderate the effect of psychological risk factors (e.g., lack of well-being, such as low happiness) on suicide behaviour [3]. Specifically, we test whether the effect of low happiness on suicide rate is moderated by statutory homelessness. A main-effects model with the focal variable low happiness and the moderator homelessness as well as the previously significant variables self-harm and children leaving care as predictors was tested against the full model extended with the moderation of happiness by homelessness (interaction effect). The statistically significant full model (Table 6) explained 45% of variance in suicide rate (*R*^2^ = 0.45), *F* (5, 145) = 32.61, *p* < 0.001. The sample results overestimated the explained variance in the outcome by 2% (*R*^2^_adjusted_ = 0.43). The main-effects model was also significant (Table 6). Crucially, we found evidence for the hypothesis: the full model explained significantly more variance (2%, Δ*R*^2^ = 0.02) in suicide rate than the main-effects model, *F* (1, 143) = 4.10, *p* = 0.045. In particular, the effect of low happiness increased as statutory homelessness decreased.

The predictor variables and the interaction effect were statistically significant at the point where they were entered into the regression, so each explained significant additional variance (*sr*^2^) in suicide rate over and above the previous predictors at their point of entry (Table 6).

#### Explanatory approach: intervention-based model

A variant of the explanatory approach is motivated by the potential for intervention to decide a priori on the predictors to include in a model. Considered are target variables that can pragmatically be influenced by potential interventions (e.g., to improve existing services or create new services) and that are (considered) causal antecedents of the outcome variable. 6^,^^7^

For instance, under consideration may be improvements of social care services to reduce social isolation among carers and social care users in order to meet their social-contact needs and to eventually reduce suicide. These improvements correspond with two variables in the suicide data set: social care users’ social-contact need fulfilment and carers’ social contact need fulfilment. We report the results of a standard (forced-entry) regression using these predictors to predict suicide. The statistically significant final model (Table 7) explained 10% (*R*^2^ = 0.10), *F* (2, 146) = 4.13, *p* = < 0.001. The sample results overestimated the explained variance in the outcome by 1% (*R*^2^_adjusted_ = .09). Both predictors were statistically significant (Table 7). As the predictors were entered at the same time, the unique variance (*sr*^2^) each explained in suicide rate was analysed rather than the additional variance explained.

**Table 7:** Standard multiple regression

Source	Unstan-dardised coefficients		Stan-dardised coefficients	*F/t*	*p*	95%-confidence interval for *b*		Correlations			Collinearity statistics	
	*b*	*SE*	β			Lower limit	Upper limit	*r*	*sr*	*sr* ^*2*^	Tolerance	VIF
Model 1				8.40	< 0.001					0.11		
Constant	1.58	0.19		8.52	.000	1.21	1.95					
Social-care users’ social-contact need fulfilment	0.01	0.00	0.22	2.70	.008	0.00	0.02	0.26	0.21	0.22	0.05	1.05
Social-care carers’ social-contact need fulfilment	0.01	0.00	0.19	2.40	.018	0.00	0.01	0.24	0.19	0.19	0.04	1.05

*R*^2^ = 0.10, *R*^2^_adjusted_ = 0.09

## Discussion

The aim of our study is to demonstrate how data analysis techniques can be used to address the issues of data reduction, prediction and explanation using online available public health data, in order to provide a sound basis for informing public health policy. In relation to this aim, our main methodological result is a set of procedures that involves reducing the set of public health indicators and analysing the importance of predictors by prediction and/or explanation. Our main substantive result is the identification of a small set of predictors of suicide rate which can be considered in public health policy-making.

Here, we first discuss the trade-off between predictive power and interpretability, followed by our results from a methodological perspective. We then proceed with a discussion of our substantive results in terms of suicide predictors. Next, we discuss informatics challenges of public health data. Finally, we present recommendations and future work regarding analysis of public health complex data from our findings.

### Trade-off between predictive power and interpretability

Our results demonstrate the need to make informed decisions about the approach to take in modelling. In the *prediction* approach, as predictors are added to the model, the model fit in terms of variance explained in the outcome will normally increase, but never decrease. However, statistical supervised-learning techniques such as multiple regression penalise the addition of poor predictors in two ways. First, poor predictors are by definition not statistically significant (e.g., as evaluated by the *t*-ratio for each regression parameter). Second, adding poor predictors reduces the improvement of predicting the outcome from the model against the inaccuracy of the model (as evaluated by the *F*-ratio).

Stepwise multiple regression (Tables 4 and 5) uses statistical rules to avoid the problem caused by adding poor predictors. However, this has two potentially undesirable consequences. As before, first, the models are less likely to be generalisable across samples [11]; in other words, models are more likely not to generalise between public health data sets. Second, the results may be difficult to interpret, as the analyst has no control over the entry of predictors and their order of entry into the final model. For example, when new predictors are added to improve model fit in analyses for prediction, existing predictors may suffer from reversal paradoxes such as suppression [45]. The remedy is to use substantive knowledge to assist in variable selection and specify a theoretically credible model [45]. Therefore, even in data analysis with automated procedures (e.g., automated construction of predictor variables, [35]), a domain expert needs to take part to ensure a meaningful analysis [37]. Moreover, Rudin [37] warns against the practice of attempts to explain ‘black-box models’ – that are seen as inherently ‘non-interpetable’ in their original form – through ‘explainable’ model versions because this ‘is likely to perpetuate bad practices and can potentially cause catastrophic harm to society’ (p. 1). Instead, the proposed solution is to create models that are interpretable to start with. Another consideration is that complex ‘black-box models’ do not necessarily always outperform simpler (interpretable) models [20].

In the *explanatory* approach, the analyst has full control over the entry of predictors and their order of entry in to the final model. In addition, the analyst has the responsibility to a priori specify a model to be tested or to specify different models to be tested against each other (Table 6). This specification is based on theory or pragmatic considerations (such as potential for intervention). The advantage of this approach is the promise of cumulative science, building on existing theory and results of theory-testing, to gain a continually increasing understanding of the outcome that is being studied (e.g., suicide) and, based on this, policy decision-making. Testing models against each other allows us to rule out certain explanations for behaviour and support other explanations. An advantage of analyses for explanation is that their results can be interpreted in the framework of relevant theories from which the models are instantiations. In contrast, the results from analysis for prediction are based on statistical criteria and therefore do not have this advantage; moreover, the results may not be generalisable.

In sum, predictive research aims to produce the most powerful model to predict outcome data from available predictor data. However, because this analysis is atheoretical it can produce results that are not generalisable and difficult to interpret. Explanatory research tests an a priori model or tests alternative models against each other, with the aim of theoretical understanding. Although this supports cumulative science and interpretability of results as a basis for policy decision-making, it does not necessarily maximise predictive power. Explanatory research is important to test theories and develop a coherent body of theoretical knowledge. In disciplines where theory is scarce and data are plentiful, predictive research can help develop causal theory as a basis for subsequent explanatory research [41].

### Methods

From a methods perspective, the main findings of our data analyses and associated considerations are as follows. The square-root and logarithmic transformations produced substantially improved distributions on the dependent variable (suicide rate) and some predictors. Moreover, data transformations substantially improved the distribution of residuals from all regression analyses. Multi-collinearity analysis was effective in identifying and subsequently removing redundant variables for multiple regression. In addition to reducing the predictor set, another benefit of multi-collinearity analysis is that, by doing this, reversal paradoxes such as suppression [45] are less likely to occur. PCA was effective in further reducing the suicide predictor variables to a three-dimensional solution with interpretable components. Although PCA and exploratory factor analysis are unsupervised learning techniques, confirmatory factor analysis [43] offers supervised learning to test the significance and generalisability factor structures. This could be beneficial to test the generalisability of, for example, higher-order predictors (such as relatedness dysfunction) of suicide in public health data.

After multicollinearity analysis, stepwise regression to predict suicide rate was effective in reducing the predictor set further to four statistically significant predictors. Stepwise regression using the component scores of principal-component analysis to predict suicide rate was effective at reducing the predictor set further to two statistically significant components. Stepwise linear regression analysis is advantageous in identifying the smallest set of predictors. Nonetheless, it requires assumptions [43], such as a linear model and normality of variable distributions, which may not be appropriate for all data sets. However, non-linear regression allows other function forms and bootstrapping provides a distribution-free alternative to significance-testing.

Other techniques to consider for reducing the predictor set include public health expert opinion in variable selection, grouping of variables into larger groups and automated statistical methods for linear model selection and regularization [20]. The latter include subset selection methods (e.g., best subset selection), shrinkage methods (e.g., ridge regression and the lasso) and ‘integrated’ dimension reduction methods (principal components regression and partial least squares). All these methods are integrated in the sense that, in contrast to data analysis in the current study, they do not separate (automated) data reduction and (automated) model testing.

Theory-based hierarchical regression for explanation was effective at establishing moderation (by statutory homelessness) of the effect of a predictor variable (low happiness) on suicide rate. Intervention-oriented standard regression for explanation was effective at establishing two significant predictors related to the universal human need of relatedness in social-care services. In addition to the assumptions of stepwise regression analysis, regression analysis for explanation also requires the analyst to specify one or more a priori models, based on domain knowledge. Expected pay-offs are model generalisability and cumulative science.

The methods that were presented in this research were specifically applied to data analysis with multiple regression. However, these methods may be applicable to statistical learning and machine learning more generally.

### Predictors of suicide

From a substantive perspective, our data analysis produced the following results and related consideration. The findings of regression analysis indicate that the evidence or history of self-harm could be used as an important indicator for targeted interventions to reduce suicide. This result supports a previous meta-analysis that established prior non-suicidal self-injury as a top-5 predictor of suicide attempt [13]. However, this is correlational evidence between suicide and self-harm at the unitary-authority level and stronger evidence would be provided if data at the individual level were available for analysis. Specifically, the prominence of self-harm as a predictor of suicide may be partially or fully an artefact. At the individual level (at which no data were available in the dataset that was analysed), suicide cases and self-harm cases may be quite distinct, with few or limited connections. For example, potentially those who commit suicide may not engage in self-harm and those who engage in self-harm often do not commit suicide.

In the intervention-based regression for explanation, both social care users’ social contact need fulfilment and carers’ need fulfilment were significant suicide predictors. These represent and provide further evidence for the universal human need of relatedness as a requirement for human thriving [40] in social care.

### The informatics challenges of public health data

While public health data have great potential to shape public health policy, there are several informatics challenges that should be considered which may introduce bias into the decision-making process or have practical implications for policy delivery. Two main challenges are (1) practical – data quality and (2) person-centred – public health leadership. Regarding data quality, the available data may be insufficiently detailed or impossible to disaggregate to allow policy decisions to be made. For example, if data related to age, gender or social class (or other moderating or mediating variables) are unavailable, targeting services for those most in need, or most likely to benefit, will be difficult to achieve. Furthermore, given the range of services that contribute to public health, integrating datasets can be difficult.

Alongside data-related issues, workforce issues also are a key component in the use of health informatics: developing effective policies through the use of public health informatics data is, ultimately, down to public health leadership. Given the increased downward pressure on public health budgets, it is necessary to improve understanding of how such data can be used among policymakers and commissioners of services, as well as the questions that public health data can (and cannot) answer as part of a wider move towards the implementation of information systems that can be used to support public health functions [9].

While public health informatics continues to expand in areas including surveillance and workforce issues, in other areas, such as communication and coordination, the field remains relatively under-developed. Without greater coordination between services and data, silos are likely to persist in public health information systems [27]. In response to the need for a more systemic approach, population health informatics is a growing topic among developed countries; population health informatics it takes a broader view and targets not only the total population (as publish health informatics does), but also target populations, provider organisations and healthcare systems [22].

### Recommendations and future work

Based on our findings, we present the following recommendations for future work. Effect size and its interpretation is an important consideration in regression modelling and classification [7]. Effect sizes should also routinely be interpreted in the analysis of suicide data and Fingertips data more generally. Moreover, minimum or worthwhile effect sizes have an important role to play as input into in statistical inference regarding obtained effects in techniques such as minimum-effect tests [30] and magnitude-based inference [19]. The use of worthwhile effect sizes as input to inference should also routinely be considered in the analysis of suicide data and public health data more generally.

Although regression techniques proved to be effective in the current study for the analysis of public health data, further data analysis techniques should be considered in future work. For predictive research, these include statistical learning techniques for prediction such as decision trees and random forests [21], support vector machines [5, 20], gradient boosting [35] and neural networks [17]. It is important to note that these techniques suffer from some of the same problems as stepwise regression analysis, in particular a potential lack of model generalisability [15] and potential lack of interpretability [46]. Moreover, because their loss functions are similar, the results of the support vector classifier and logistic regression can often be highly similar [20]. To consider in explanatory research are also techniques such as minimum-effect tests [30], magnitude-based inference [19] and Bayesian regression [24]. These further techniques can complement or replace regression techniques, depending the aim of data analysis.

Specifically, first, mediation analysis can be used to provide evidence for the causal process (the ‘why’) of the treatment effect [18, 26]. To gain a better understanding of public health outcomes (e.g., suicide) from a process perspective, analysts should identify potential mediators in their models and then conduct appropriate mediation analysis. For example, further analysis can be carried out, using self-harm as a mediator, to better understand the factors influencing self-harm and thereby indirectly suicide.

Moderation analysis can be used to provide evidence the conditions under which (when) a treatment effect exists [18]. To gain a better understanding of public health outcomes (e.g., suicide) from the perspective of boundary conditions, analysts should identify potential moderators in their models and then conduct appropriate moderation analysis (see, e.g., Table 6). The combination of mediation and moderation analysis (conditional process analysis; [18]) can provide further insights in the conditions (moderation) under which mechanisms (mediation) that explain (suicide or other) outcomes operate. For example, this analysis can establish whether the mediated effect of a suicide prevention intervention is moderated by baseline score (the conditions under which mediation occurs).

Second, future work could use time-series data analysis to identify local authorities that have shown (positive or negative) significant change in suicide rate in recent years. Recommendations could then be made to conduct field work to investigate the causes of this change and possible interventions.

Third, our data analysis was at the level of local authority. However, the predictors of health-public outcomes may vary across different levels of analysis (for example, general medical practice, unitary authority, and region). Therefore, future work should identify available data at different levels and analyse the data accordingly in an integrated fashion through multi-level analysis [8], allowing exceedingly complex models to be tested.

In the analysis of suicide behaviour, the currently available data set allows no meaningful analysis of indicators together with demographic indicators. This is because, first, a breakdown by demographics (gender, age) was not available for some indicators. Second, the breakdown was inconsistent among the remaining variables (e.g. different age brackets were used for different indicators). Therefore, the current analysis did not include demographics. Accordingly, a recommendation for future data collection is that data are consistently broken down by demographics and recorded in public health data sets.

Public health interventions to reduce suicide (e.g., men’s sheds; [49]) may influence outcomes (e.g., suicide rate). However, the current data sets do not include information about interventions (e.g., type of intervention, target population, duration). Future work should therefore collect data on interventions and integrate these with the data that are already collected, in a way that facilitates evidence-based analysis of theory-based interventions [29].

## Conclusions

We have demonstrated how regression techniques can be effective in the analysis of online complex public health data. We showed how these techniques can be beneficial to analyse suicide data in terms of data reduction, and modelling for prediction and explanation. The method for data analysis carried out in this study, based on a small fraction of the vast array of available public health data, can extend beyond suicide prevention to aid the understanding and interpretation of public health data and hence develop better strategies to improve the health of the public.

## Additional files

Additional file 1: Transforming the data. **Table S1.** Skewness and kurtosis of suicide profile variables. (ZIP 21 kb)

Additional file 2: Assessing bias. **Figure S1.** Plot of standardised predicted values against standardised residuals. **Figure S2.** Histogram and P-P plot of standardised residuals. (ZIP 91 kb)

Additional file 3: **Table S2.** Multiple regression for transformed and original variables. (XLSX 11 kb)

## Data Availability

The dataset and the data analysis are available at http://sssl-staffweb.tees.ac.uk/u0011128/Fingertips.data.and.analysis/R.zip and http://sssl-staffweb.tees.ac.uk/u0011128/Fingertips.data.and.analysis/SPSS.zip. The dataset can also be accessed via the R package fingertipsR (https://cran.r-project.org/web/packages/fingertipsR/index.html).

## Abbreviations

PCA: Principal-component analysis
VIF: Variance inflation factor

## Footnotes

1 “a set of tools for modeling and understanding complex datasets” ([20], p. vii). We use the term ‘statistical learning’ rather than ‘machine learning’ as the former more accurately represents the statistical analysis used in this paper.

2 “rich source of indicators across a range of health and wellbeing themes that has been designed to support JSNA [Joint Strategic Needs Assessment] and commissioning to improve health and wellbeing, and reduce inequalities.” [34]

3 Excluded were the Isles of Scilly, City of London and Rutland because of limited data availability.

4 To allow for comparison of between the different approaches, the results of variance accounted for (R^2^) are presented for each approach.

5 In hierarchical and stepwise regression, the semi-partial correlation coefficient squared denotes additional variance explained by each predictor in the outcome variable, but in forced-entry regression it denotes unique variance explained.

6 Please note that the Fingertips repository does not contain data about interventions. Instead, here we analyse variables that could be targeted by interventions.

7 Potential covariates (such as the predictors from a stepwise regression model) that cannot be (directly) influenced by the intervention under consideration are not included in the model.
