# Supplementary material for: Explainable statistical learning in public health for policy development: the case of real-world suicide data
Source: BMC Med Res Methodol. 2019 Jul 17;19:152. doi: 10.1186/s12874-019-0796-7 (PMC6636096; doi:10.1186/s12874-019-0796-7)
Supplement: Supplementary file 1 — Transforming the data. Table S1. Skewness and kurtosis of suicide profile variables. (ZIP 21 kb) [file 12874_2019_796_MOESM1_ESM.zip › Additional file 1.docx]

# Additional file 1: transforming the data

We analysed the distribution shape of each variable as well their z-scores of their kurtosis (peakedness) and skewness statistics (Tabachnick & Fidell, 2013). The outcome measure was suicide rate from the 2014 Fingertips Suicide Prevention Profile data. We analysed suicide predictor variables from the most recent previous years (2013 where available or otherwise from the most recent year before 2013) (see Table S1).

The analysis of the outcome variable ‘suicide rate’ and 30 potential predictor variables (Table S1; histograms available on request) shows that a majority of variables suffered from a poor distribution in terms of skew and/or kurtosis (|z-score| > 3). We applied three typical transformations (square root, logarithm and inverse) on these variables to improve their distributions. After inspection of the results, a final decision was made to apply a logarithmic transformation to 15 variables, a square transformation to 3 variables and an inverse to 1 variable. After the final transformation, absolute standardised skewness had reduced below 3 for all variables and absolute standardised kurtosis for all but one variable (Table S1). Subsequent data analyses used the transformed variables.
