# Supplementary material for: Explainable statistical learning in public health for policy development: the case of real-world suicide data
Source: BMC Med Res Methodol. 2019 Jul 17;19:152. doi: 10.1186/s12874-019-0796-7 (PMC6636096; doi:10.1186/s12874-019-0796-7)
Supplement: Supplementary file 2 — Assessing bias. Figure S1. Plot of standardised predicted values against standardised residuals. Figure S2. Histogram and P-P plot of standardised residuals. (ZIP 91 kb) [file 12874_2019_796_MOESM2_ESM.zip › Additional file 2.docx]

# Additional file 2: assessing bias

In analysing any regression model, an assessment of bias is important to establish its generalisability (Field, 2017). This includes the analysis of casewise diagnostics and assumptions (Field, 2017). We illustrate this assessment with the final model from the analysis presented in Table 4. Regarding casewise diagnostics, standardised residuals (SR) are analysed in comparison with the standard normal distribution. Accordingly, 5% of SRs should lie beyond ±2 and 2.5% beyond ±2.5. The first condition was met, with 8 (5%) SRs beyond ±2. The second condition was also met, with 2 (1%) SRs beyond ±2.5. Therefore, the residuals show a lack of bias.

Cook’s distance is an indicator of each predictor’s potential undue influence on the model. With all absolute distance values below 1, none of the cases were deemed problematic. Leverage and the Mahalanobis distance are secondary measures of influence of cases over the predicted values (Field, 2017), and the distance is also a measure of multivariate-outlier status (Tabachnick & Fidell, 2013). Leverage values should be within 2 or 3 times the theoretical average leverage. Here, the average value was (k+1)/n = (4+1)/149 = 0.03. With leverage values of 0.91 to 1.14, six cases (125, 146, 100, 1, 3 and 9) exceeded the leverage cut-off and were therefore potentially influential over the predicted values. Mahalanobis values should be below the critical χ^2^-value at p = 0.001 (Tabachnick & Fidell, 2013), in this case χ^2^(2, crit) = 18.47. With χ^2^(4) = 18.49 and 20.68, Cases 3 and 9, respectively exceeded this cut-off; therefore, again these cases were potentially influential and also multivariate outliers, but their standardised residuals were not extreme (z = 0.42 and -0.16, respectively). Case 9 was also unusual in terms of extremely high scores on both PCA Components 1 and 2 (z = 2.72 and 2.86, respectively).

Standardised DFBeta is an indicator of each case’s influence on the model parameters (regression coefficients). Here, none of the absolute DFBeta values exceeded the cut-off of 1; therefore, none of the cases had an undue influence. The covariance ratio is a measure of each case’s influence on the variance of the regression parameters. Here, the ratio values of six cases (129, 103, 47, 113, 26, 139) were below the lower cut-off (0.94 = 1 - [3(2+1)/149] = 1 - [3(k+1)/n]); therefore, deleting these cases would improve the precision of some of the model parameters (Field, 2017). The ratio value of eight further cases (130, 125, 101, 22, 100, 146, 3, 9) exceeded the upper cut-off (1.06) = 1 + [3(2+1)/149] = 1 + [3(k+1)/n]); therefore, deleting these cases would damage the precision of some of the model parameters (Field, 2017).

In sum, the analysis generally showed a lack of bias. Six cases may be further investigated as potentially influential over the predicted values and two cases as multivariate outliers. The cases with covariance ratio below the lower limit may also be investigated further as they potentially influence the variance of regression parameters.

The assumption of homoscedasticity and linearity was assessed by comparing the standardised residuals against the predicted outcomes scores (Figure S1). This analysis shows evidence of homoscedasticity, with, first, a roughly equal spread of standardised residuals across the range of standardised predicted values. The analysis also demonstrated linearity, with a zero slope for the predicted values against the residuals. The assumption of normality of the residuals was assessed by histogram, and skewness and kurtosis. The histogram and the P-P plot showed an approximately normal-distribution shape (Figure S2) and skew and kurtosis values were excellent (zS = 0.08) and satisfactory (zK = 0.84), respectively. Moreover, none of the standardised residuals were beyond ±3, demonstrating a lack of outliers in the solution.
